# Surgeon posture evaluation during simulated laparoscopic and robotic-assisted cholecystectomy

**DOI:** 10.1007/s11548-026-03666-4

**Published:** 2026-05-21

**Authors:** Jakob Reinhardt, Max Bergholz, Luca Wegener, Jonas Fuchtmann, Rebecca Rack, Klaus Bengler, Dirk Wilhelm

**Affiliations:** 1 Technical University of Munich, School of Engineering and Design, Munich, Germany; 2 Technical University of Munich, School of Medicine and Health, Munich, Germany

**Keywords:** Robotic-assisted surgery, Human factors, Ergonomics, Posture analysis

## Abstract

**Purpose:**

Robotic camera systems have been developed to assist surgeons by holding the endoscope during laparoscopic cholecystectomies, a task traditionally performed by a human assistant. While such systems relieve the assistant from an ergonomically unfavorable task, there are concerns about the effects their usage has on the surgeon’s posture. Addressing these concerns requires the use of posture assessment methods that are compatible with the constraints of surgical settings.

**Methods:**

This feasibility study compares two simulated cholecystectomies from one surgeon: one performed with robotic endoscope assistance and one without. A scaled medical mockup designed for laparoscopic procedures and a certified robotic endoscope holder (Soloassist 2, AKTORmed GmbH) were applied in an experimental operating room. Two motion capture systems (VICON and Xsens MVN Awinda) were used to capture motion and posture data of the surgeon.

**Results:**

The analysis suggests that the surgeon maintains a similar, largely static posture in both scenarios. Despite minor anomalies, the observed postures are predominantly ergonomically correct. Consequently, under the examined conditions, the use of robotic assistance does not support the hypothesis of either conferring ergonomic advantages or disadvantages for surgeons.

**Conclusion:**

While posture evaluations have traditionally relied on eyewitness observation or questionnaires, this article proposes an exploratory analysis based on acquired motion data. While evidence from a single surgeon is not sufficient to draw generalizable conclusions, the presented approach demonstrates how motion data can be used to objectively assess surgeon posture during laparoscopic and robotic-assisted surgery.

## Introduction

### Ergonomic challenges in laparoscopic and robotic-assisted surgery

The use of robotic-assisted surgery (RAS) is becoming increasingly common in various laparoscopic surgical procedures. Advances lie in an improved human–machine interaction in terms of enhanced precision and control for the surgeon  [1]. However, while improving some aspects of ergonomics, RAS may also introduce new challenges. The positioning of the robot must be harmonized with the patient’s anatomy and the working space of the staff. Often, the resulting ergonomic implications of RAS on the surgeons’ posture remain a concern. The spatial demands of robotic systems and their incorporation into the surgical workflow can impose ergonomically unfavorable postures on surgeons [2, 3].

The motion behavior and joint angles of the surgeon during RAS are critical factors influencing overall posture and comfort. Laparoscopic and robotic-assisted procedures are characterized by prolonged periods of relatively static work, during which sustained postures of poor ergonomics can be particularly harmful to musculoskeletal health [4, 5]. A bent trunk and extended arms lead to problems if endured for extended periods [6, 7]. Consequently, back and shoulder pain are among the most commonly reported musculoskeletal issues by surgeons [4, 8].

Bending the trunk forward/backward may be classified in terms of one of four load zones. The first zone corresponds to the optimal working posture, where the trunk bends forward up to 20$^{\circ }$. The second zone includes bending between 20$^{\circ }$ and 60$^{\circ }$ forward. When the trunk bends forward beyond 60$^{\circ }$ or bends backward, the risk of developing musculoskeletal disorders increases rapidly. Comfortable working posture also requires an arm angle of less than 20$^{\circ }$ in flexion and extension. Between 20$^{\circ }$ and 60$^{\circ }$ and especially above shoulder level, the risk of musculoskeletal disorders increases [9].

The Rapid Upper Limb Assessment (RULA) is a widely used observational method for evaluating upper-body posture in occupational settings [17]. RULA scores individual body segments according to their angular deviation from neutral and combines them into a single action-level score. In addition, RULA applies a binary penalty of +1 whenever a posture is held statically for longer than one minute. While this 60 s threshold provides a practical decision rule, it treats a 61 s hold identically to a hold of several minutes and assigns no penalty at all to sustained postures just below the threshold. In surgical procedures, where prolonged static postures are common, this step function approach may fail to capture the continuous nature of fatigue accumulation.

Most results in the literature focus on reported internal strain and musculoskeletal disorders in laparoscopic surgery. Quantification of external stress or posture, and how these findings may differ in the context of RAS, is underrepresented. Traditionally, posture evaluations have been conducted through eyewitness observation, and internal strain has been assessed using questionnaires; however, these methods can be subjective and prone to bias and misses [10, 11]. Hence, there is a need for more objective and reliable quantitative methods to assess surgeon ergonomics.

### Surgeons’ task and robotic integration

Most surgeries are clustered into three phases: preoperative, operative, and postoperative. In the preoperative phase, the patient is prepared for surgery, including anesthesia and positioning. With the first incision, the operative phase begins. Postoperative, the patient is extubated and transferred to the wake-up room. Within this evaluation, a cholecystectomy was used as a representative minimally invasive intervention. The procedure combines typical tasks of laparoscopic surgery and has a high degree of standardized steps. For these reasons, this procedure has often been used as a benchmark in the literature [12, 13]. In Germany with 161,734 cases in 2018, the procedures is one of the most frequently performed laparoscopic surgeries [14]. A cholecystectomy begins with an initial exploration of the abdomen, followed by preparation of the surrounding tissue. The relevant vessels are identified and subsequently clipped and transected. The gallbladder is then dissected from the liver bed and removed.

While in the preoperative and postoperative phases the surgeon is moving around the operating table and reaching for instruments, the operative phase is characterized by more static work. Risks during the operative phase of laparoscopic surgery include limitations on table positioning imposed by the type of procedure and patient geometry, constraints on monitor placement due to limited available space, and restricted instrument handling resulting from small incision sites [15]. When a robot is introduced, the surgeon must additionally adapt to the robot’s workspace and control interface. Although the robot offers high precision, its geometric footprint is less flexible than that of a human assistant.

Research has often focused on multi-arm systems like the daVinci Surgical System (Intuitive Surgical, Inc.) [16]. In this work, we focus on smaller single-arm robotic systems that can hold and can be controlled to manipulate the endoscope, such as the Soloassist 2 (AKTORmed GmbH). To optimize the integration of such technology, it is necessary to investigate the specific differences in surgeon behavior across the various phases of surgery.

## Methodology

### Research questions

The aim of this study is to investigate:

1. Whether the surgeon’s posture and motion differ across the three phases of the surgery.
2. Whether unfavorable postures are maintained for extended periods of time.
3. Whether the surgeon’s posture and motion differ between robot-assisted surgery (RAS) and conventional laparoscopic surgery.
4. Whether the applied motion tracking and analysis method is suitable for capturing and analyzing the surgeon’s posture during surgery.

Posture is operationalized by analyzing the surgeon’s trunk and shoulder flexion–extension angles during surgery. Motion is operationalized by analyzing the surgeon’s trunk and hand movements.

### Surgical environment and procedures

**Fig. 1:**
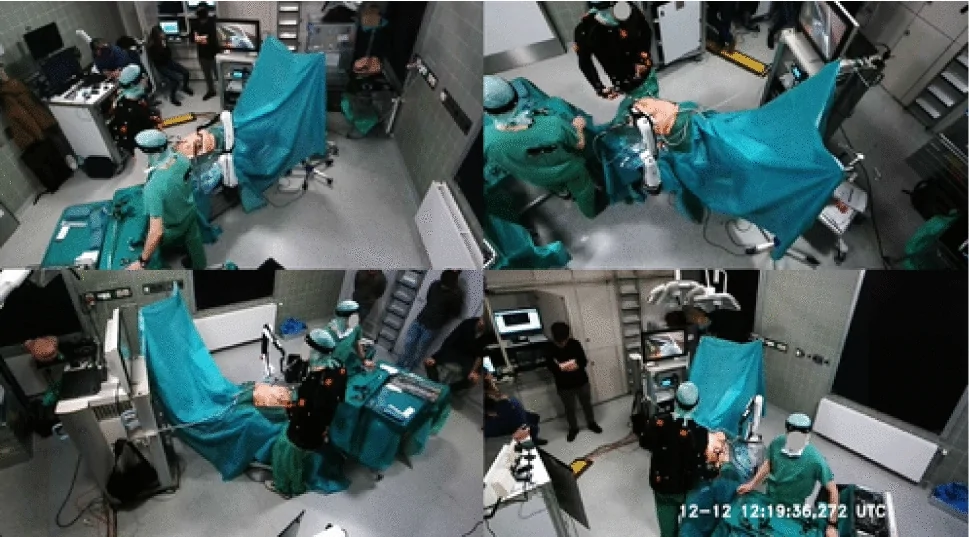
Frames extracted from continuous video camera recordings of the surgical scene in the RAS scenario in the experimental operating room. The scene is captured simultaneously from four different perspectives

The study was conducted in the experimental operating room at the Technical University of Munich University Hospital (TUM Klinikum) (Fig. 1). The surgeon is an experienced visceral surgeon. He performs two simulated surgeries: One is a conventionally laparoscopic cholecystectomy (conventional), and the other one is a robotic-assisted laparoscopic cholecystectomy (RAS). In the case of conventional surgery, the surgeon is assisted by a surgical assistant and a surgical technician. In the scenario of RAS, the surgical assistant is replaced by the robot. The surgeon controls the robot, and consequently the endoscopic view, using a small joystick mounted on the right hand. The setup includes standard surgical instruments, a screen, and the certified Soloassist 2 (AKTORmed GmbH) robotic endoscope holder. A scaled medical mockup designed for laparoscopic procedures was placed on the patient table. The model is made of specialized rubber and includes predefined trocar insertion points. Inside the model, a handcrafted gallbladder and surrounding tissue were positioned. To enable further qualitative analysis, the surgery was recorded on video from four different perspectives.

Both simulated procedures were structured into three phases: preoperative, operative, and postoperative. The preoperative phase began with the initiation of model preparation (positioning, draping, and instrument setup). The preoperative phase concluded with the initial incision at one of the designated trocar insertion points. The operative phase covered the period from the first incision through completion of the surgical task (exploration, tissue preparation, clipping, transection, and gallbladder removal). The phase ended the moment the gallbladder was removed from the model. The postoperative phase started at operative completion and included wound closure and simulated patient handoff. Phase transitions were annotated in real time by a trained observer at the moment of the aforementioned predefined surgical tasks. In addition, the start and end of robotic assistance were recorded as part of the operative phase. Because this was a simulated procedure performed on a medical mockup rather than a live patient, the duration of each phase is likely shorter than in a clinical setting. The total procedure durations (Table 1) should therefore be interpreted accordingly. Data collection spanned the entire duration from preoperative initiation to postoperative completion. The data remain suitable for within-subject comparison of posture across phases and surgical approaches, but do not represent real-world operative times.

### Motion capture systems

Two motion capture systems were employed: a marker-based VICON system (Vicon Motion Systems Ltd., UK) and an IMU-based Xsens MVN Awinda system (Xsens Technologies B.V., Netherlands).

The infrared-based Vicon MX T-Series system comprised nine infrared cameras mounted to the ceiling using a dedicated camera truss. The operating surgeon wore a black Velcro motion capture suit (Qualisys AB, Sweden) equipped with 42 spherical infrared reflective markers, positioned according to the University of Southampton Kinematic Model. Ten Xsens IMU sensors were attached to the upper body and calibrated in accordance with the manufacturer’s instructions to record upper-body posture. Data from both systems were collected at a sampling rate of 60 Hz.

Since motion trajectories are the primary output of the VICON system, the surgeon’s movement behavior was analyzed using VICON data. Conversely, as joint angles constitute the primary output of the Xsens system, the surgeon’s joint kinematics were analyzed using Xsens data.

### Data analysis and results

Vicon data were processed using the Nexus software suite (version 2.14). Marker positions were initially reconstructed using the software’s reconstruction algorithm. Unidentified marker trajectories were subsequently labeled manually. Gaps in the trajectories were filled by estimating missing segments based on the trajectories of identified markers within the same rigid body segment.

**Fig. 2:**
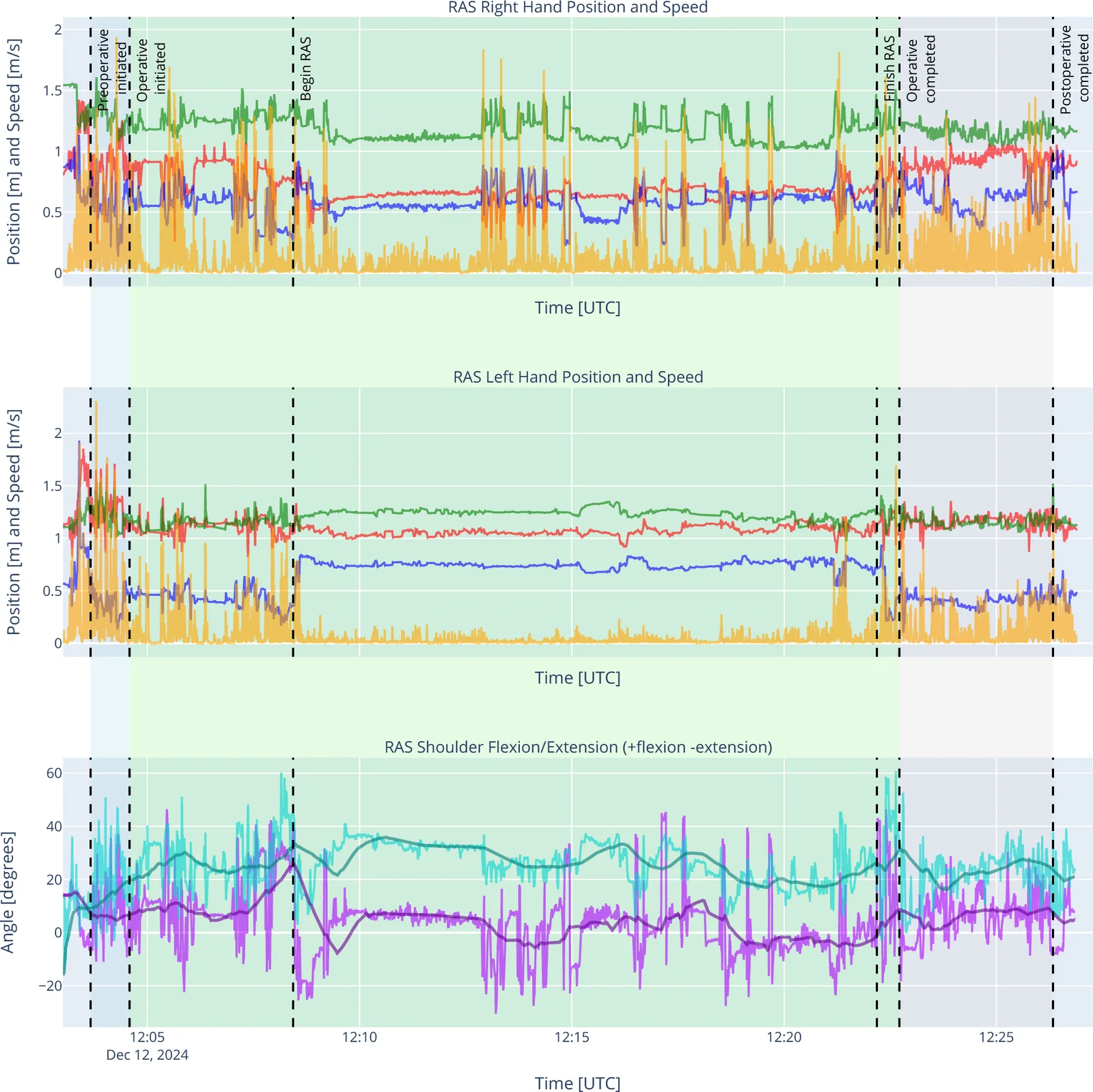
Time-series data of the surgeon’s hand positions and speeds, as well as shoulder flexion–extension angles, during robotic-assisted surgery. Vertical dashed lines indicate changes in surgical phases and the points at which the robot is engaged. The markers correspond to the following events: “preoperative initiated” marks the start of patient preparation; “operative initiated” indicates the first incision; “begin RAS” and “finish RAS” denote the period during which the robotic endoscope holder was actively in use (a subset of the operative phase); “operative completed” marks the end of the surgical task; and “postoperative completed” indicates the end of wound closure and simulated patient handoff. Line descriptions: top: right hand coordinates x (red), y (blue), z (green), hand speed (orange); Middle: Left hand coordinates x (red), y (blue), z (green), hand speed (orange); Bottom: Right shoulder angle (light purple), right shoulder angle rolling mean 60 s (dark purple), left shoulder angle (light turquoise), left shoulder angle rolling mean 60 s (dark turquoise)

Velocity data were derived from positional data using a five-point stencil differentiation method. For exploratory analysis, line plots were generated for the movement of each body part and the joint angles. Figure 2 presents the time-series data of hand movements and shoulder angles for the robotic-assisted surgery (RAS) case. All plots for both the conventional and RAS trials are available as supplementary material (https://jakobreinhardt.github.io/CARS2026).

The T8 marker, located at the center of the back, was used to analyze trunk position and movement speed. Right and left hand markers were used to quantify hand/arm movements. For joint angle analysis, the pelvis-T8 angle was used to assess trunk posture, while corresponding Xsens shoulder measurements were used to capture right and left shoulder flexion and extension.

Table 1 summarizes the average motion and posture metrics of the surgeon during the three phases of the surgery for RAS and conventional setup.

**Table 1:** Motion and posture metrics of the surgeon during the three phases of surgery for both RAS and conventional setups

Metric	Procedure	Preoperative phase	Operative phase	Postoperative phase
Duration	Conventional	206 s	1093 s	196 s
Duration	RAS	55 s	1088 s	217 s
Speed T8 (trunk)	Conventional	0.08 m/s (0.12 m/s)	0.04 m/s (0.07 m/s)	0.05 m/s (0.08 m/s)
Speed T8 (trunk)	RAS	0.12 m/s (0.12 m/s)	0.03 m/s (0.06 m/s)	0.05 m/s (0.09 m/s)
Angle pelvis-T8 (trunk)	Conventional	− 5.53$^{\circ }$ (5.16$^{\circ }$)	3.00$^{\circ }$ (5.16$^{\circ }$)	7.61$^{\circ }$ (4.28$^{\circ }$)
Angle Pelvis-T8 (trunk)	RAS	− 10.59$^{\circ }$ (4.10$^{\circ }$)	− 9.48$^{\circ }$ (3.14$^{\circ }$)	−9.83$^{\circ }$ (5.25$^{\circ }$)
Speed right hand	Conventional	0.20 m/s (0.23 m/s)	0.10 m/s (0.16 m/s)	0.09 m/s (0.16 m/s)
Speed right hand	RAS	0.29 m/s (0.25 m/s)	0.11 m/s (0.18 m/s)	0.16 m/s (0.18 m/s)
Speed left hand	Conventional	0.20 m/s (0.25 m/s)	0.05 m/s (0.09 m/s)	0.07 m/s (0.11 m/s)
Speed left hand	RAS	0.31 m/s (0.32 m/s)	0.05 m/s (0.10 m/s)	0.11 m/s (0.14 m/s)
Angle right shoulder	Conventional	10.20$^{\circ }$ (10.96$^{\circ }$)	0.90$^{\circ }$ (9.56$^{\circ }$)	2.88$^{\circ }$ (7.66$^{\circ }$)
Angle right shoulder	RAS	7.78$^{\circ }$ (10.23$^{\circ }$)	3.98$^{\circ }$ (12.54$^{\circ }$)	5.97$^{\circ }$ (7.74$^{\circ }$)
Angle left shoulder	Conventional	1.95$^{\circ }$ (8.91$^{\circ }$)	− 5.21$^{\circ }$ (9.66$^{\circ }$)	− 14.34$^{\circ }$ (5.85$^{\circ }$)
Angle left shoulder	RAS	20.58$^{\circ }$ (11.05$^{\circ }$)	26.84$^{\circ }$ (8.35$^{\circ }$)	21.93$^{\circ }$ (7.95$^{\circ }$)

Values are presented as mean (± standard deviation), except for duration, which is given as an absolute value

#### Differences in posture and motion across surgical phases

Figure 2 shows that the preoperative and postoperative phases are characterized by increased movement, such as reaching for instruments or moving around the operating table, whereas the operative phase is largely static. This is reflected in the average speeds: The trunk and hands exhibit the highest speeds during the preoperative phase, all body parts reach a minimum during the operative phase, and a slight increase is observed in the postoperative phase (Table 1).

In the conventional case, the average trunk angle shifts from slight flexion to slight extension across the three phases, with only minor changes between phases. In the RAS case, the average trunk angle remains around 10$^{\circ }$ flexion throughout all three phases.

For the shoulder angles, higher average values are observed during the preoperative and postoperative phases compared to the operative phase, likely reflecting short reaching and grasping movements. During the operative phase, both hands remain relatively static, as indicated by the hand velocity data and shoulder angles. This static posture corresponds to the precise and controlled movements required for laparoscopic instrument handling, during which the arms are maintained in a low, stable position.

#### Posture duration

Traditional RULA applies a penalty if a pose is statically held for over one minute. To provide a more differentiated view of postural exposure, we extend this concept by defining several time window categories that reflect different levels of behavioral and ergonomic relevance.

**Fig. 3:**
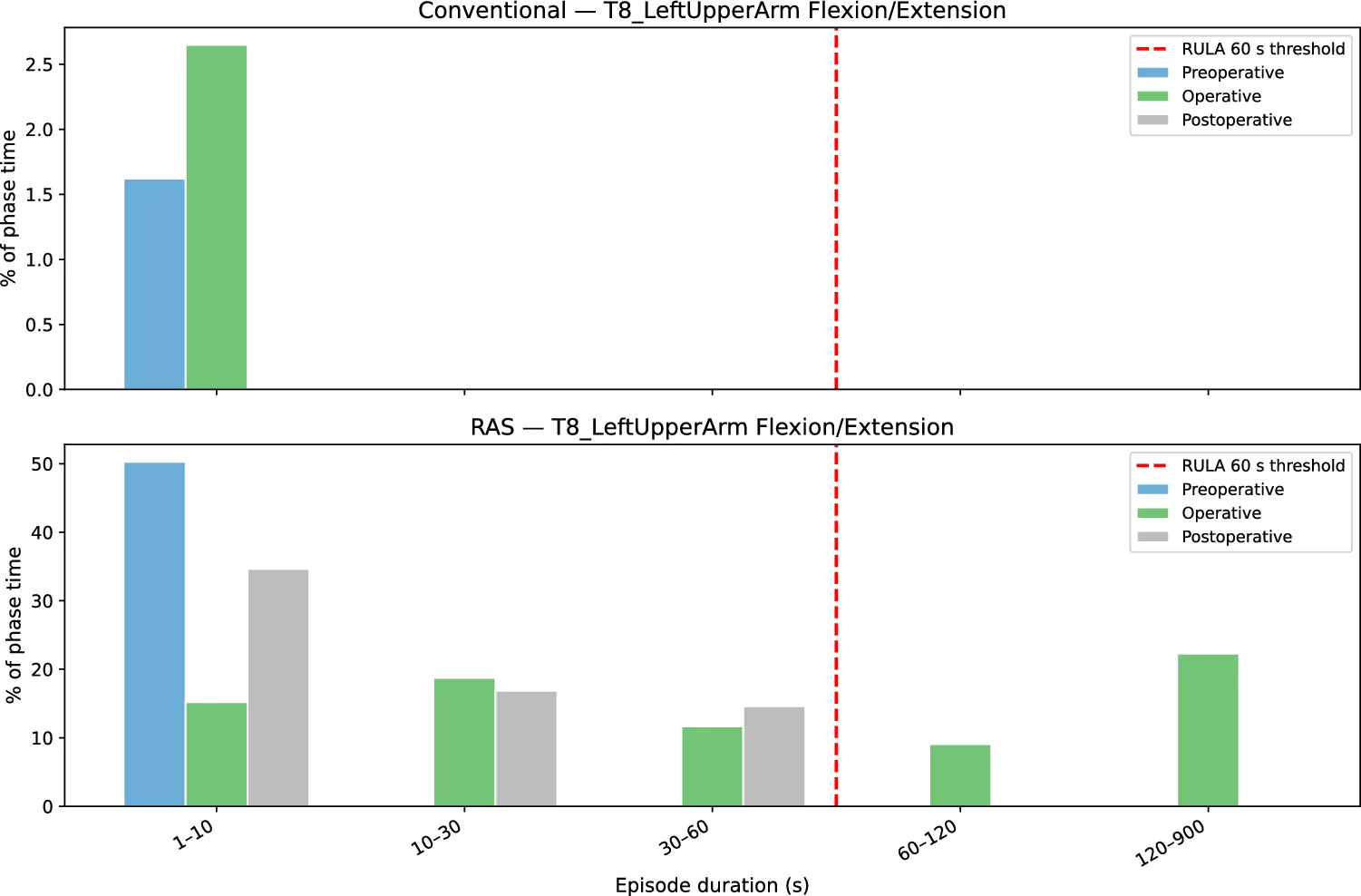
Grouped bar chart showing the percentage of total phase time that the surgeon spent in unfavorable postures (>20$^\circ$) for left shoulder flexion and extension, broken down by episode duration bin. Episodes shorter than 1 s are excluded to suppress measurement noise. The bins are 1–10 s (brief lapses), 10–30 s (short deviations), 30–60 s (sustained postures below the RULA threshold), 60–120 s (postures exceeding the threshold), and 120–900 s (prolonged exposure). The dashed red line marks the 60 s boundary at which RULA assigns its +1 penalty. Top panel: conventional surgery; bottom panel: robotic-assisted surgery (RAS)

To quantify how long specific postures were maintained, we identified all consecutive episodes during which the joint angle exceeded 20$^{\circ }$ and grouped them into duration bins. Episodes shorter than 1 s were excluded to suppress measurement noise. The remaining episodes were assigned to five bins of increasing duration: 1–10 s (brief posture lapses, common during dynamic task execution), 10–30 s (short deviations from neutral posture), 30–60 s (sustained postures approaching the one-minute RULA threshold), 60–120 s (postures exceeding the RULA threshold), and 120–900 s (prolonged exposure associated with increased fatigue and risk of musculoskeletal disorders [5]). For each bin, the total episode time was expressed as a percentage of the respective phase duration, so that the bars indicate what fraction of a given surgical phase was spent in unfavorable postures of a particular duration class. The dashed red line in Fig. 3 marks the 60 s boundary at which RULA assigns its binary penalty, highlighting the contrast between the continuous distribution of episode durations and RULA’s step function approach.

In the left shoulder example (Fig. 3), the operative and postoperative phases in the RAS scenario exhibit a notable share of time in the 1–10 s bin, indicating frequent but brief deviations from neutral posture. Sustained episodes of 30–60 s appear predominantly during the operative phase, while episodes exceeding the 60 s RULA threshold are observed almost exclusively in the operative phase of the RAS scenario. The conventional scenario shows a different pattern with lower overall percentages spent in unfavorable postures. This is consistent with the lower average shoulder flexion noted in Table 1. Remarkably, only short lapses of unfavorable angles are observed in the conventional scenario.

#### Posture comparison between conventional and RAS

In the RAS scenario, a conspicuity is observed in the posture of the left shoulder. The left shoulder flexion angle remains at approximately 25$^{\circ }$ for an extended period (Fig. 2), resulting in comparatively high average shoulder flexion values (Table 1). Comparison with the video recordings suggests that the surgeon maintained a slightly rotated upper-body posture, which in turn led to sustained flexion of the left arm.

In addition, the two scenarios exhibit distinct arm movement characteristics. While both trials involve reaching movements, as indicated by subjective observation, the RAS scenario shows a noticeably higher fraction of phase time spent in brief posture lapses that exceed 20$^{\circ }$ (1–10 s) compared with the conventional trial.

#### Motion tracking and analysis method

A visual inspection of the raw motion data (Fig. 2 and supplementary material) revealed outliers and times of remarkable postures during the surgery. Aggregated metrics (Table 1) facilitate comparison between different surgical phases and scenarios. The posture duration bar chart (Fig. 3) enables quantification of the fraction of phase time spent in unfavorable postures of different duration classes. For validation of conspicuousness, the video recordings were viewed as validation. The combined use of the VICON and Xsens systems, together with video recordings, enabled a comprehensive analysis of the surgeon’s movement behavior.

## Discussion

Among the three phases of surgery, the operative phase occupies the greatest proportion of time. This phase involves the use of laparoscopic instruments within a very confined workspace. One might therefore expect an increase in static work or potentially poor postures due to constrained positioning around the operating table compared to the other surgical phases. Overall, the surgeon’s posture remains predominantly within recommended ergonomic guidelines with one notable exception. The raw data revealed prolonged flexion of the left shoulder. Visual inspection of the video recordings showed a slight torsion of the upper body during the RAS operative phase, likely resulting in sustained left shoulder flexion as a compensatory posture. This upper-body torsion may be attributed to the fixed positioning of the robotic system, which can restrict the surgeon’s ability to adjust posture optimally. Notably, this flexion was detectable only through the collected raw data and would likely have gone unnoticed if assessment had relied solely on video footage or eyewitness observation.

Poor posture was operationalized using measures of trunk flexion and extension, as well as shoulder flexion and extension. However, the captured data offer additional perspectives that were not within the scope of this work. The full set of joint angles could be further leveraged by integrating them into standardized ergonomic assessment tools, such as the RULA score [17]. While RULA simplifies interpretation by combining multiple joint angles into a single harmonized score, this aggregation reduces data granularity and reflects its original design for production and logistics environments rather than surgical tasks. Moreover, RULA’s binary time-based penalty, which uniformly penalizes static postures held longer than 60 s, lacks the sensitivity to capture the varying durations and intermittent nature of sustained postures encountered during surgery. This contrasts with the approach used here, which emphasizes an in-depth analysis of the acquired raw data rather than collapsing it into a composite metric.

Limitations include the possibility that the markers may have hindered the surgeon. For instance, we observed occasional collisions between instrument-mounted markers and the robotic arm. Additionally, the heat generated by the motion tracking suit may have been distracting, potentially contributing to postures that were not fully representative of typical surgical behavior. Regarding potential inaccuracy in the sensor-derived joint angle data, the manufacturer of the XSens system reports root mean square (RMS) differences of less than 5$^{\circ }$ for the primary joint angles during walking. To assess absolute sensor drift, they conducted a study with 10 participants and found no evidence of drift over a 90-minute recording period [18].

This study is based on data from a single surgeon performing two simulated procedures, and the findings should therefore be regarded as exploratory. Although cholecystectomy is a highly standardized procedure with uniform task protocols, surgeon posture is also influenced by individual anthropometrics, experience level, and personal movement strategies. Consequently, the observed posture patterns may not generalize across surgeons. Future studies should include multiple surgeons of varying body sizes and experience levels to provide more robust evidence.

This article proposes a motion data analysis approach for ergonomic posture assessment in robotic-assisted surgeries and provides one of multiple viewpoints, necessary to evaluate surgical workflows with the goal to improve the integration of robots in surgical workflows. Besides posture analysis, other perspectives such as cognitive load and efficiency should be considered to evaluate RAS on a more holistic level.

## Conclusion

This feasibility study demonstrates an exploratory and quantitative perspective on the analysis of the surgeon’s posture during laparoscopic and robotic-assisted surgery. The analysis of motion behavior and joint angles provides insights into the ergonomic challenges faced by surgeons during RAS. The findings suggest that certain phases of the surgery are more static than others. In the presented case, these are the phases where the robot is applied. This highlights the need for careful ergonomic considerations when integrating robotic assistance systems. In addition, the surgical phases are characterized by distinct motion patterns and postures. Despite minor anomalies, the observed postures are predominantly ergonomically appropriate. Given that this study is based on a single surgeon and simulated procedures, the findings should be interpreted as exploratory. Nevertheless, this approach illustrates how motion data can be leveraged to objectively evaluate surgeon posture during laparoscopic and robotic-assisted procedures.

The field of quantifying surgeon ergonomics remains relatively young and rapidly evolving. As surgical procedures and robotic technologies continue to advance, dedicated and adaptable methods are required to accurately capture and interpret both surgical workflows and robotic integration. Evaluating robotic-assisted surgery demands multiple complementary perspectives, with the goal of improving the holistic integration of robotics within surgical workflows.

## Data Availability

The datasets generated and analyzed during the study are displayed in the following repository: https://jakobreinhardt.github.io/CARS2026.
